# Humanized VHH-hFc Fusion Proteins Targeting the L-HN Fragment of Tetanus Toxin Provided Protection In Vivo

**DOI:** 10.3390/antib14020048

**Published:** 2025-06-13

**Authors:** Yating Li, Kexuan Cheng, Jiazheng Guo, Yujia Jiang, Qinglin Kang, Rong Wang, Peng Du, Chen Gao, Yunzhou Yu, Zhixin Yang, Wei Wang, Jiansheng Lu

**Affiliations:** 1 Laboratory of Advanced Biotechnology, Beijing Institute of Biotechnology, Beijing 100071, China; lyt18214729367@163.com (Y.L.); 16696653458@163.com (K.C.); sdqzgjz@163.com (J.G.); jiangyujia19990627@163.com (Y.J.); kql_lynn@163.com (Q.K.); wangrong_8312@163.com (R.W.); dependable@zju.edu.cn (P.D.); chengao2015@163.com (C.G.); yunzhouyu@163.com (Y.Y.); yy_xiao@126.com (Z.Y.); 2 Department of Occupational Health and Occupational Diseases, College of Public Health, Zhengzhou University, Zhengzhou 450001, China

**Keywords:** tetanus toxin, TL-HN, VHH, phage-display library, humanized VHH-hFc fusion protein, neutralization

## Abstract

Background: Tetanus toxin, produced by *Clostridium tetani*, is the second deadliest known toxin. Antibodies capable of neutralizing tetanus toxin (TeNT) are vital for preventing and treating tetanus disease. Methods: Herein, we screened thirty-six single variable domains on a heavy chain (VHHs) binding to the light chain (L) and the translocation domain (HN) (L-HN) fragment of TeNT from a phage-display library. Then, the L-HN-specific clones were identified, humanized, and fused with a human fragment crystallizable region (hFc) to form humanized VHH-hFc fusion proteins. Results: The humanized VHH-hFc fusion proteins TL-16-h1-hFc, TL-25-h1-hFc, and TL-34-h1-hFc possessed potent efficacy with high binding affinity, specificity, and neutralizing activity. Only 0.3125 μg was required for TL-16-h1-hFc or TL-25-h1-hFc, and 0.625 μg was required for TL-34-h1-hFc to provide full protection against 10 × Lethal Dose 50 (LD_50_) TeNT. In the prophylactic setting, 125 μg/kg of TL-16-h1-hFc or TL-25-h1-hFc provided full protection even when they were injected 12 days before exposure to 10 × LD_50_ TeNT, while TL-34-h1-hFc was less effective. In the therapeutic setting, 25 μg/kg of TL-16-h1-hFc or TL-25-h1-hFc could provide complete protection when administered 24 h after exposure to 5 × LD_50_ TeNT, while TL-34-h1-hFc required 50 μg/kg. Conclusion: Our results suggest that TL-16-h1-hFc, TL-25-h1-hFc, and TL-34-h1-hFc provide a bright future for the development of anti-TeNT preventive or therapeutic drugs.

## 1. Introduction

Tetanus is a devastating disease caused by tetanus neurotoxin (tetanus toxin, TeNT), characterized by major symptoms such as muscle rigidity, spasms, spastic paralysis, respiratory deficits, and autonomic dysfunction [1,2,3]. Tetanus has an extremely high morbidity and mortality rate, with neonatal tetanus death rates approaching 100% in the absence of medical intervention and a global mortality rate of 30–50% even with aggressive combination therapy, making it an extremely serious and potentially fatal disease [4,5].

Tetanus toxin, a potent neurotoxin, is synthesized by *Clostridium tetani* as a single 150 kDa protein, composed of a light chain (L, 50 kDa), a heavy chain (H, 100 kDa), and an interchain disulfide bond that is critical for neurotoxicity. Depending on its different functions, the H chain is subdivided into a C-terminal domain (Hc, 50 kDa) and an N-terminal domain (HN, 50 kDa) [6]. The Hc domain is responsible for neuronal cell binding and endocytosis into vesicles [7,8,9]. The HN domain facilitates the translocation of the L chain into the neuronal cytoplasmic lysate [6,9,10]. The L domain functions as a zinc-dependent protease and is connected to the HN domain through a critical interchain disulfide linkage. It cleaves the neuronal soluble N-ethylmaleimide-sensitive factor attachment protein receptor (SNARE) protein vesicle-associated membrane protein 2 (VAMP2), leading to inhibitory neurotransmitter release and subsequent generalized muscle spasms [10,11,12,13,14].

Although tetanus can be prevented by vaccination with a tetanus toxoid (TT), tetanus still causes high mortality worldwide [15]. It remains a major killer, especially in countries that do not implement adequate public health measures [16]. *Clostridium tetani* infection does not confer lifelong immunity; therefore, emergency tetanus immunization is needed after trauma to provide effective and immediate intervention to prevent tetanus [17,18]. Passive immunization agents commonly used in clinical practice include tetanus antitoxin (TAT) and human tetanus immunoglobulin (TIG). However, TAT and TIG have significant limitations: TAT suffers from a long immunization cycle and potential hypersensitivity of the receptor [19,20], and TIG carries the risk of transmitting known and unknown infectious diseases and is difficult to obtain [21,22]. Therefore, there is an urgent need for a suitable and safe alternative that can be expressed in large quantities in vitro.

Different from traditional monoclonal antibodies, single variable domains on a heavy chain (VHHs), also termed nanobodies (Nbs), comprise heavy-chain antibodies naturally occurring in Camelidae [23]. These VHHs have the characteristics of a small relative molecular mass, strong tissue penetration, and high stability and specificity [24,25,26]. However, the advantages of VHH might also be their shortcomings. Their small size enables good tissue penetration and distribution but also facilitates their rapid removal by the kidneys [25]. Conjugation with a human fragment crystallizable region (hFc) has been identified as a viable option to prolong the half-life of a VHH and enhance its neutralizing ability to a certain extent [27].

Herein, we aimed to develop humanized VHHs to neutralize TeNT. We identified three specific VHHs (TL-16, TL-25, and TL-34) from a phage-display VHH library constructed by immunizing camelids using the L-HN fragment of tetanus toxin as the antigen. By fusion with hFc and humanization through amino acid sequence substitution, the humanized VHH-hFc fusion proteins (TL-16-h1-hFc, TL-25-h1-hFc, and TL-34-h1-hFc) retained the potent efficacy of the parental VHHs in terms of their high binding affinity, specificity, and neutralizing activity and showed potential to be used as preventive and therapeutic proteins.

## 2. Materials and Methods

### 2.1. Reagents, Cell Lines, and Animals

The recombinant proteins TeNT, TL-HN (L-HN fragment of TeNT), TL (L domain of TeNT), THN (HN domain of TeNT), THC (Hc domain of TeNT), and rTeNT (full-length TeNT) were prepared in a previous laboratory study [28,29] by expression in *Escherichia coli* and purification using the AKTA Explorer system after induction by Isopropyl βD-1-thiogalactopyranoside (IPTG). Siglec-15 protein was purchased from Beijing Yiqiao Shenzhou Technology Co. (Beijing, China). AL-HN, BL-HN, EL-HN, and FL-HN are the L-HN domains of botulinum toxin types A, B, E, and F, which were prepared by our laboratory as controls [30,31,32,33].

FreeStyle™ HEK293-F cells (Thermo Fisher Scientific, Waltham, MA, USA) were maintained in FreeStyle™ 293 Expression Medium at 37 °C in a humidified atmosphere with 5% CO_2_ for the production of the hFc fusion protein.

A healthy adult male Bactrian camel was acquired from Inner Mongolia, China. Female KM mice (specific pathogen-free, 18–20 g) were purchased from Beijing Spefo Biotechnology Co. (Beijing, China) and housed under a controlled 12 h light/12 h dark cycle with unrestricted food and water.

### 2.2. Immunization of the Camel

Purified TL-HN was emulsified with an equal volume of Floyd’s Complete Adjuvant (Sigma-Aldrich, St. Louis, MO, USA). The emulsion was administered to a healthy adult Bactrian camel via multi-site injections at 14-day intervals. Freund’s Incomplete Adjuvant (Sigma-Aldrich, St. Louis, MO, USA) was used for all booster immunizations following the initial dose. Serum samples were collected after four immunization rounds. The specific antibody titer against TL-HN in camel serum was quantified by enzyme-linked immunosorbent assay (ELISA) (Corning, New York, NY, USA). A fifth immunization was performed after it was determined that a high titer of antibody had been obtained. Seven days after the last immunization, 150 mL of camel blood was collected, and lymphocytes were separated using lymphocyte isolation solution.

### 2.3. Construction of the Phage-Display Nanobody Library

Total RNA was extracted from lymphocytes and reverse transcribed into cDNA. The VHH coding fragments were amplified by two rounds of PCR and cloned into the phage expression vector (ScFv-NEN). The ligated products were electrotransformed into *E. coli* TG1 cells, which were inoculated on 2YT-GA agar plates and incubated inverted overnight at 37 °C. Single colonies were selected and their DNA sequenced to determine whether the VHH coding fragments were inserted into the vectors. The correct insertion rate and the diversity of the library were assessed based on the sequencing results.

### 2.4. Biopanning and Isolation of the TL-HN-Specific VHHs

To screen the specific phage clones, the TL-HN protein was encapsulated on immunization tubes using 20 μg and 10 μg of protein for the first and second rounds of selection, respectively. The immunization tubes were sealed with a sealing solution (phosphate-buffered saline (PBS) containing 100 μg/mL bovine serum albumin) and incubated for 2 h at room temperature. Subsequently, the phage-display library was added to the immunization tubes and incubated for 2 h at room temperature. The immunization tubes were rinsed with PBS and PBST (PBS containing 0.5% Tween-20), and then, 1 mL of elution buffer (0.1 M glycine-HCl, pH 2.2) was added to elute the bound phage. Then, the eluted phage was mixed with *E. coli* TG1 cells grown to the logarithmic phase. After incubation at 37 °C for 30 min and shaking at 150 rpm for 30 min, the mixture was centrifuged (2000× *g*, 10 min) at room temperature, followed by resuspension of the cells in fresh medium, clone counting, and preparation of phage-display VHHs libraries. After two rounds of panning, the single phage colonies were prepared for subsequent specific clone identification. The phage count for each panning round represents the input, while the number of clones obtained after each round reflects the output. The ratio of input to output for each round was calculated to determine the enrichment factor.

After two rounds of screening, the TL-HN protein was used as the antigen for Phage-ELISA. Positive clones specifically targeting TL-HN were obtained, and their sequences were analyzed.

### 2.5. Construction, Expression, and Purification of VHH-hFc Fusion Protein

The positive clones were used as templates to amplify the gene fragments of the VHHs, which were then inserted into the expression vector pTSE-hFc containing the Fc fragment of the human antibody. The constructed VHH-hFc fusion protein expression plasmids were sequenced to ensure the correct sequence.

The plasmids were transfected into FreeStyle^TM^ HEK293-F cells, and the cell culture supernatant was harvested and purified using the AKTA Pure Purification System (GE Healthcare, Piscataway, NJ, USA). The VHH-hFc fusion antibody was purified from the cell culture supernatants using a HiTrap MabSelect Xtra purification column (Cytiva, Uppsala, Sweden) and eluted using citrate solution (100 mM, pH 3.0). The fusion proteins were then purified using a desalting column (Cytiva), with the buffer exchanged to citrate solution (10 mM, pH 6.0). The molecular weight of the VHH-hFc fusion protein was confirmed by SDS-PAGE.

### 2.6. Cross-Reaction and Binding Activity of the VHH-hFc Fusion Proteins

The concentrations of recombinant TL-HN and other antigens were adjusted to 2 μg/mL using 50 mM carbonate solution, followed by addition to a 96-well plate at 100 μL per well and overnight incubation at 4 °C. The plates were washed six times with PBST, and then, 3% skimmed milk powder was added for blocking at 37 °C for 2 h. After washing the plate six times with PBST, the VHH-hFc fusion protein was added at 100 μL/well and incubated at 37 °C for 1.5 h. After washing with PBST, 100 μL/well of horseradish peroxidase (HRP)-labeled goat anti-human IgG (1:4000) was added and incubated at 37 °C for 45 min. The plate was washed six times with PBST, and then, o-Phenylenediamine (OPD) color development substrate was added to develop the color. The reaction was terminated by adding 50 μL of 2 M H_2_SO_4_ per well, and the absorbance was measured at 492 and 630 nm using a microplate reader (Molecular Devices, San Jose, CA, USA).

In addition, the VHH-hFc fusion proteins were diluted to 0.2 μg/mL in 2-fold dilutions for a total of 14 gradients, and the other steps were performed as described above. The relative binding of the subject’s antibody to the antigen was determined as the effective concentration (EC_50_), and curve fitting was performed using GraphPad Prism software 8 (version 8.0.2.263, GraphPad Inc., San Diego, CA, USA).

### 2.7. KD Analysis and Cross-Competitive Binding Assay

Binding of TL-16-hFc, TL-25-hFc, and TL-34-hFc to TL-HN was measured using the ForteBIO^®^ Octet QK^e^ System (Pall ForteBio Corporation, Menlo Park, CA, USA), based on BioLayer Interferometry (BLI). The VHH-hFc fusion proteins diluted to 200 nM with Hepes buffered saline-extended (HBS-EP) buffer were immobilized on Anti-hIgG Fc Capture (AHC) biosensors. After performing a 1 min baseline assay in HBS-EP buffer, the biosensor was immersed into the sample wells containing a series of gradient dilutions of TL-HN for correlation for approximately 6 min. This was followed by a 6 min dissociation step in HBS-EP buffer. The equilibrium dissociation constant (KD) values were calculated using a 1:1 binding model in Data Analysis Software 7.0 (Pall ForteBio Corporation).

Competitive binding assays were also performed to determine whether the three VHH-hFc fusion proteins recognized the same epitope on the surface of the TL-HN. One of the VHH-hFc fusion proteins was diluted to 200 nM using HBS-EP Buffer (Cytiva) and immobilized on the AHC sensor. After 1 min of determining the HBS-EP baseline, an association step was performed with the TL-HN diluted to 400 nM in HBS-EP buffer. Subsequently, a re-association step was performed in a new sample plate containing 200 nM TL-16-hFc, TL-25-hFc, TL-34-hFc, or HBS-EP Buffer. The results were analyzed using a 1:1 binding model and ForteBio Data Analysis Software 7.0.

### 2.8. Neutralizing Activity

To assess the neutralizing activity of the VHH-hFc fusion proteins, female KM mice weighing 18–20 g (four mice per group randomly) were intraperitoneally (i.p.) injected with 0.5 mL of a dilution buffer (50 mM KH_2_PO_4_, 50 mM Na_2_HPO_4_, 1 M NaCl, and 1% gelatin) containing 10 × LD_50_ of TeNT, with or without 5 μg of the VHH-hFc fusion protein. Before injection, the solution was incubated at 37 °C for 30 min. Mice treated with the TeNT and dilution buffer mixture served as negative controls. Mice were monitored daily for signs of tetanus or mortality over a 7-day period, allowing for preliminary screening of VHH-hFc fusion proteins with neutralizing activity against TeNT.

The VHH-hFc fusion proteins showing good neutralizing activity were further tested in a dose-dependent manner to calculate their neutralization titers. The VHH-hFc fusion proteins were diluted into five different doses (1.25 μg, 0.625 μg, 0.3125 μg, 0.156 μg, and 0.078 μg) and mixed with 10 × LD_50_ of TeNT, making a total volume of 500 μL. The mixtures were pre-incubated at 37 °C for 30 min, and the other steps followed the same protocol as the preliminary screening.

### 2.9. Humanization Modification and Activity Evaluation of VHH-hFc Fusion Proteins

To develop humanized VHH-hFc fusion proteins, amino acid sequences were analyzed using the Abysis database (http://www.abysis.org/abysis/, accessed on 16 January 2025). Framework residues with a frequency below 0.1 were substituted with high-frequency *Homo sapiens* residues based on Z-score analysis to enhance humanization. The 3D structure of the humanized IgG was modeled using Swiss-Model, and solvent-accessible surface area analysis was performed to identify residues suitable for humanization

Humanized sequences were synthesized, and the PTSE-VHH-HFc expression vector was constructed and transfected into FreeStyle™ 293-F cells to express the humanized VHH-hFc fusion proteins (TL-16-h1-hFc, TL-25-h1-hFc, and TL-34-h1-hFc). The affinity of the three humanized VHH-hFc fusion proteins was determined using ELISA, BLI, and KD analysis. The neutralizing abilities of the VHH-hFc fusion proteins were likewise examined using the mouse model in a neutralization assay. In addition, the humanized VHH-hFc fusion proteins were grouped two by two according to their binding epitopes. The neutralizing activity of the combination of two humanized VHH-hFc fusion proteins was measured using mouse neutralization experiments.

### 2.10. Preventive and Therapeutic Effects of the Humanized VHH-hFc Fusion Proteins in a Mouse Model

To assess the preventive effect of the humanized VHH-hFc fusion proteins, female SPF KM mice (18–20 g) were randomly divided into groups (*n* = 4 per group), and the VHH-hFc fusion protein was administered via the tail vein of the mice at 25 μg/kg or 125 μg/kg, with TL-34-h1-hFc injected at 50 or 250 μg/kg. Negative controls were injected with PBS, controls were injected with B-h3 (a VHH-hFc fusion protein targeting BoNT/B) as an irrelevant control, and positive controls were injected with TIG. TeNT (10 × LD_50_) was injected intraperitoneally at 12 h, 24 h, 48 h, 3 d, 5 d, 7 d, 9 d, 12 d, or 14 d, respectively, and the mice continued to be observed for 7 days.

To assess the therapeutic effect of the humanized VHH-hFc fusion protein after exposure to TeNT, 5 × LD_50_ TeNT was injected intraperitoneally into female KM mice. After 1 h, 3 h, 6 h, 12 h, or 24 h of exposure to TeNT, mice were administered the VHH-hFc fusion protein via the tail vein (25 μg/kg or 125 μg/kg, with TL-34-h1-hFc injected at 50 or 250 μg/kg). Positive and irrelevant controls included TIG and B-h3, respectively. The health status of the mice was monitored continuously, and any fatalities were recorded over a 7-day period.

### 2.11. Statistical Analysis

Data from the prophylactic and therapeutic efficacy studies were analyzed using GraphPad Prism. Statistical significance of protection differences compared to the control group was assessed with the log-rank test, with *p*-values < 0.05 considered significant.

## 3. Results

### 3.1. Construction and Biopanning of the Phage-Display VHH Library

A Bactrian camel was immunized with the purified TL-HN as the antigen, and camel serum was collected before and after immunization and subjected to ELISA analysis. The results indicate that the antibody titer increased significantly after immunization. Peripheral blood mononuclear cells (PBMCs) were isolated to extract the total RNA encoding the VHH fragments. The amplified VHH coding fragments were ligated into expression vectors and electrotransformed into *E. coli* TG1 cells to construct a library containing the VHH fragments. The capacity of the bacterial library exceeded 3.1 × 10^8^ colony forming units (CFU).

Panning was performed using TL-HN as the antigen. To obtain more specific and high-affinity clones in this second round, we reduced the coating concentration of purified TL-HN from 20 μg to 10 μg. After two rounds of screening, the ratios of the output to the input increased to 8.4 × 10^−5^ and 1.95 × 10^−4^, respectively (Table 1).

Positive clones that could bind to TL-HN after the second round of elution were selected using phage-ELISA, with bovine serum albumin as an antigenic control. From 200 phage clones, 166 TL-HN positive binders were identified and sequenced, and the sequences of the VHHs were analyzed using the IMGT/V-QUEST webpage. Based on the differential sequences of complementarity-determining region 3 (CDR3), 36 sequences were finally obtained, which were named TL-1 through TL-36.

### 3.2. Expression and Purification of VHH-hFc Fusion Proteins

**Figure 1:**
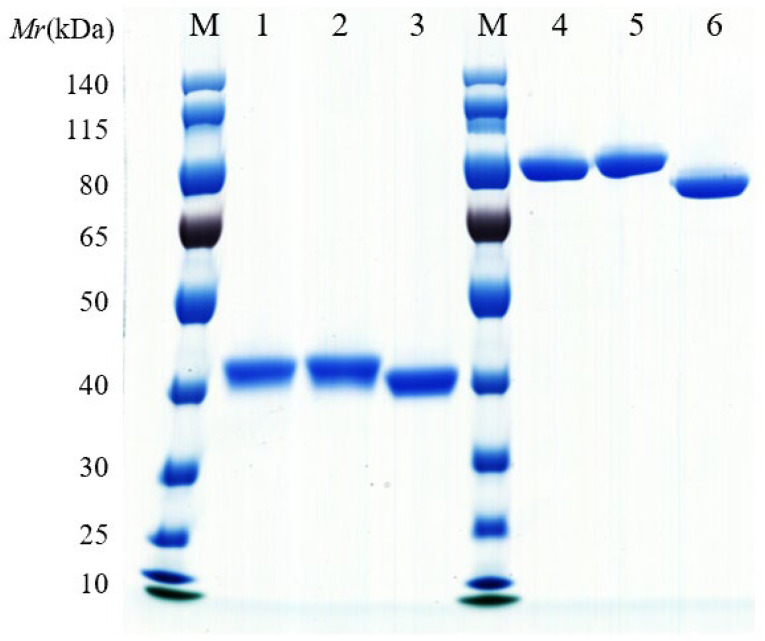
Evaluation of the VHH-hFc fusion proteins using SDS-PAGE. (**left**) SDS-PAGE under reducing conditions. Lane 1, TL-16-hFc; lane 2, TL-25-hFc; lane 3, TL-34-hFc. (**right**) SDS-PAGE under non-reducing conditions. Lane 4, TL-16-hFc; lane 5, TL-25-hFc; lane 6, TL-34-hFc; M, protein markers.

The coding sequences of TL-1 to TL-36 were inserted into the expression vector pTSE-hFc containing the human Fc fragment coding sequence to express the VHH-hFc fusion proteins. They were then expressed in FreeStyle^TM^ HEK293-F cells and purified using the AKTA Pure Purification System. The molecular weights of the VHH-hFc fusion proteins were determined using SDS-PAGE. The results show that these VHH-hFc fusion proteins were 40 KDa under reducing conditions and 80 kDa under non-reducing conditions, respectively, corresponding to the theoretical molecular weights of the VHH-hFc fusion proteins (Figure 1).

### 3.3. Preliminary Screening of VHH-hFc Fusion Proteins with Effective Neutralizing Activity In Vivo

The neutralizing activity of all VHH-hFc fusion proteins was evaluated by challenging KM mice with TeNT. In each group, four mice were challenged with 10 × LD_50_ of TeNT pre-incubated with either 5 μg of each VHH-hFc fusion protein or dilution buffer. All control mice treated with dilution buffer died within 3 days. Three VHH-hFc fusion proteins (TL-16-hFc, TL-25-hFc, and TL-34-hFc) provided complete protection, and the remaining VHH-hFc fusion proteins showed incomplete protective efficacy. The results indicated the great potential neutralizing potency of these three VHH-hFc fusion proteins against TeNT (Table 2).

### 3.4. Binding Activity and Cross-Reactivity of the VHH-hFc Fusion Proteins

**Figure 2:**
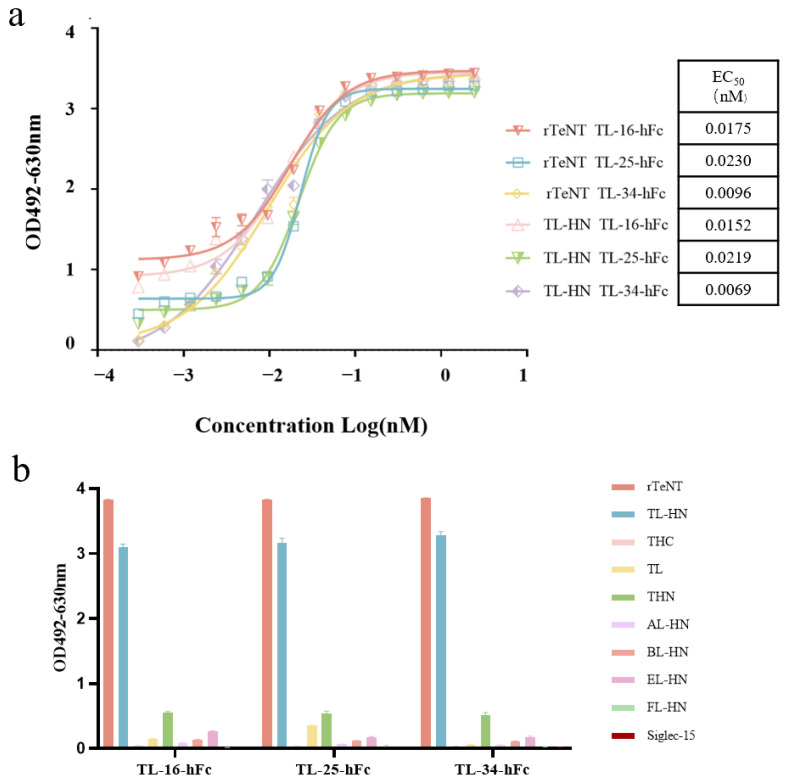
Evaluation of the basic properties of the VHH-hFc fusion proteins. (**a**) Binding assay of the VHH-hFc fusion proteins to rTeNT or TL-HN proteins using ELISA. The concentration of the antigens was 2 μg/mL, and that of VHH-hFc fusion proteins was initially 0.2 μg/mL, followed by 2-fold dilutions for a total of 14 gradients. (**b**) The specificity of TL-16-hFc, TL-25-hFc, and TL-34-hFc. The binding between the VHH-hFc fusion proteins and different antigens. The concentrations of the antigens were all 2 μg/mL, and the concentration of the VHH-hFc fusion proteins was 0.1 μg/mL.

We carried out ELISA to determine the binding ability of the VHH-hFc fusion proteins to TL-HN. All VHH-hFc fusion proteins bound to TL-HN in a concentration-dependent manner. The results indicated that the three VHH-hFc fusion proteins showed strong binding activity to TL-HN, with EC_50_ values ranging from 0.006 to 0.025 nM (Figure 2a).

The specificity of the VHH-hFc fusion proteins was detected using ELISA. The results showed that the VHH-hFc fusion proteins bound specifically to rTeNT and TL-HN but did not cross-react with the other antigens (Figure 2b).

### 3.5. Affinity and Competitive Binding Activity of the VHH-hFc Fusion Proteins

To analyze the affinity of the VHH-hFc fusion proteins more accurately, BLI was performed to determine the affinities of the three VHH-hFc fusion proteins for TL-HN. According to the data in Table 3, all VHH-hFc fusion proteins bound to TL-HN with high affinity, with KD values ranging from 3 nM to 11 nM.

**Figure 3:**
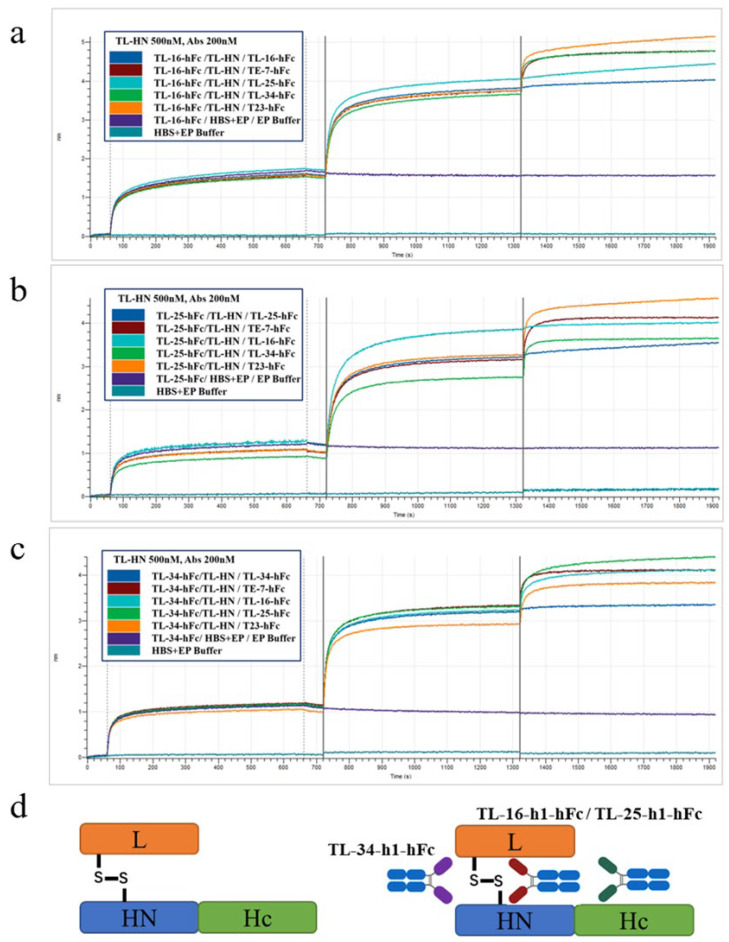
Competitive binding analysis of three VHH-hFc fusion proteins using BLI. (**a**–**c**) Kinetic processes of the three antibodies TL-16-hFc, TL-25-hFc, and TL-34-hFc binding to the TL-HN protein in different orders; in the legend box, samples separated by a slash “/” represent the “Loading,” “Association,” and “Re-association” stages of the sample molecules. TE-7-hFc and T23-hFc serve as control groups. (**d**) Structural diagram of TeNT and the mapped epitopes of TL-16-hFc, TL-25-hFc, and TL-34-hFc on the TL-HN fragment in the competitive binding experiment.

To detect whether the VHH-hFc fusion proteins target different antigenic regions of TL-HN, a competitive binding assay was performed. The results showed that there was competition between TL-16-hFc and TL-25-hFc, suggesting that they might target the same epitope of TL-HN (Figure 3).

### 3.6. Neutralizing Activity of VHH-hFc Fusion Proteins at Different Doses

**Figure 4:**
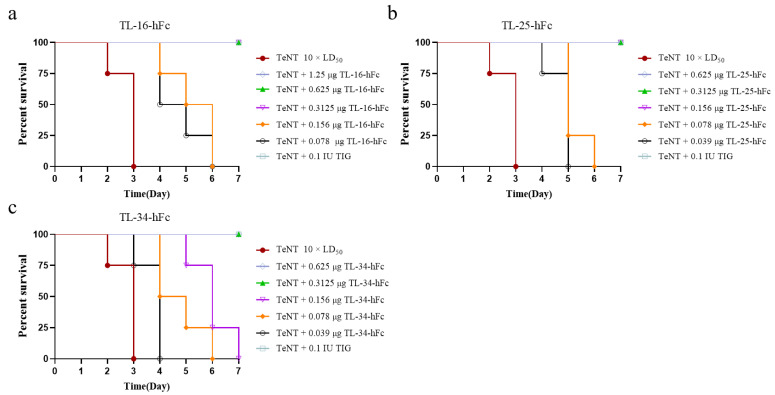
Evaluation of the neutralization efficiency of the VHH-hFc fusion proteins. The VHH-hFc fusion proteins were diluted into five different doses and mixed with 10 × LD_50_ of TeNT. Then, the mixture was injected intraperitoneally into mice, with four mice in each group. (**a**–**c**) The time of death and the number of surviving mice were observed once a day, and the percentage of surviving mice was plotted for each VHH-hFc fusion protein.

We further evaluated the neutralizing activity of TL-16-hFc, TL-25-hFc, and TL-34-hFc against TeNT in KM mice. After diluting the VHH-hFc fusion proteins to five different doses (1.25 μg, 0.625 μg, 0.3125 μg, 0.156 μg, and 0.078 μg) and pre-incubating them with 10 × LD_50_ TeNT, 500 μL of the mixtures were injected into each mouse intraperitoneally. The negative control comprised TeNT mixed with diluent. A standard antitoxin of TeNT was used as a positive control. The results showed that 0.3125 μg of TL-16-hFc or TL-34-hFc could completely protect the mice against 10 × LD_50_ TeNT, and TL-25-hFc was even more effective, requiring as little as 0.156 μg (Figure 4).

### 3.7. Humanization and Characterization of the Humanized VHH-hFc Fusion Proteins

To reduce the immune response, the three camel-derived VHHs were humanized according to analysis at the website http://www.abysis.org/abysis/ (accessed on 16 January 2025) and Swiss-model. Humanization of the three VHH-hFc fusion proteins was successfully completed after sequence analysis and non-critical amino acid substitutions.

**Figure 5:**
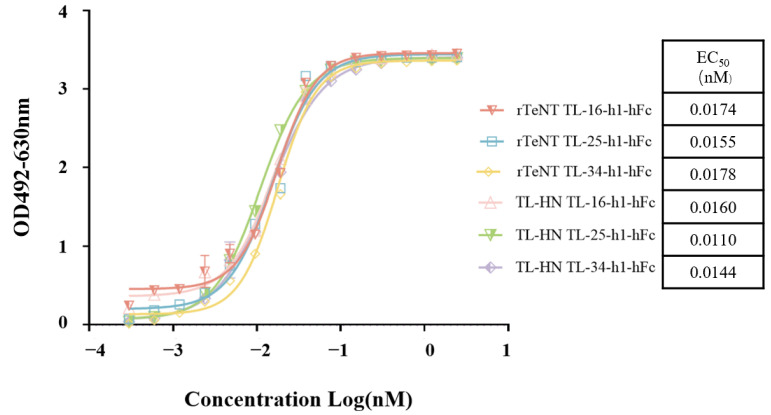
ELISA binding assay of the humanized VHH-hFc fusion proteins to rTeNT or TL-HN proteins. The concentration of rTeNT or TL-HN was 2 μg/mL, and that of the VHH-hFc fusion proteins was initially 0.2 μg/mL, followed by 2-fold dilutions for a total of 14 gradients.

We used ELISA to estimate the binding ability of the humanized VHH-hFc fusion proteins. The EC_50_ values of the three humanized VHH-hFc fusion proteins were between 0.011 nM and 0.018 nM (Figure 5). To further analyze the affinity of the humanized VHH-hFc fusion proteins, the KD was measured using BLI. The KDs of TL-16-h1-hFc, TL-25-h1-hFc, and TL-34-h1-hFc were 0.9094 nM, 0.573 nM, and 0.633 nM, respectively (Table 4). Thus, the binding ability of the three humanized VHH-hFc fusion proteins was increased compared with the non-humanized proteins.

**Figure 6:**
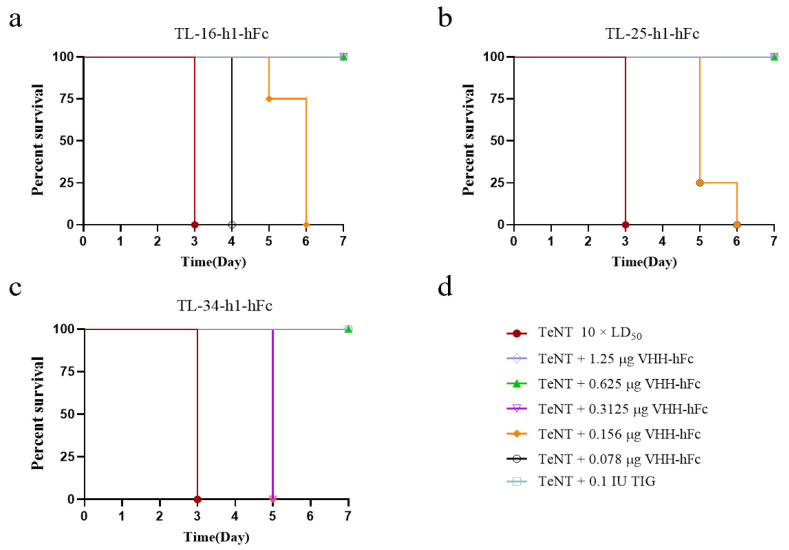
Evaluation of the neutralization efficiency of VHH-hFc fusion proteins. (**a**–**c**) Survival curves for TL-16-hFc, TL-25-hFc, and TL-34-hFc fusion proteins, respectively. (**d**) The three VHH-hFc fusion proteins were diluted to 1.25 μg, 0.625 μg, 0.3125 μg, 0.156 μg, and 0.078 μg and then mixed with 10 × LD_50_ TeNT3.8. Prophylactic and Therapeutic Efficacy of Humanized VHH-hFc Fusion Proteins In Vivo.

In addition, the neutralization abilities of the humanized VHH-hFc fusion proteins were assessed. Although the humanized VHH-hFc fusion proteins also provided protection in mice, their neutralizing ability was reduced compared with that of the non-humanized proteins. Thus, 0.3125 μg was required for TL-16-h1-hFc and TL-25-h1-hFc to fully protect against 10 × LD_50_ TeNT, and 0.625 μg was required for TL-34-h1-hFc to provide full protection (Figure 6).

TL-16-h1-hFc and TL-25-h1-hFc target a similar epitope of TL-HN; therefore, we combined two of the three humanized VHH-hFc fusion proteins. As shown in Table 5, the combination of TL-25-h1-hFc and TL-34-h1-hFc showed the best protection, while TL-16-h1-hFc and TL-25-h1-hFc showed the worst protection. Although TL-16-h1-hFc and TL-25-h1-hFc were the two most neutralizing antibodies when used alone, they did not protect well in combination, confirming that TL-16-h1-hFc and TL-25-h1-hFc target the same or a similar epitope.

Protection experiments were performed in a mouse model. The positive and negative controls were TIG and PBS, respectively, and B-h3 served as an irrelevant control.

Mice were injected with different doses of proteins and PBS via the tail vein at different time points before intraperitoneal injection with 10 × LD_50_ TeNT. The irrelevant antibody B-h3 and PBS had no preventive effect. As shown in Table 6, mice pretreated with 250 μg/kg of TL-34-h1-hFc received complete protection within 7 days. Moreover, 125 μg/kg of TL-16-h1-hFc or TL-25-h1-hFc provided full protection to mice within 12 days. Mice pretreated with 50 μg/kg of TL-34-h1-hFc were fully protected within 24 h before exposure to 10 × LD_50_ TeNT. While at a dose of 25 μg/kg, TL-16-h1-hFc provided 50% protection within 3 days, and TL-25-h1-hFc provided 75% protection within 5 days. Mice pretreated with the proteins showed reduced symptoms or delayed death. These results indicate that TL-16-h1-hFc, TL-25-h1-hFc, and TL-34-h1-hFc could protect against TeNT and that their protective effects were dose- and pretreatment-dependent.

To further evaluate the therapeutic efficacy of the humanized VHH-hFc fusion proteins, different doses of proteins or PBS were injected i.v. at 1 h, 3 h, 6 h, 12 h, or 24 h after exposure to 5 × LD_50_ TeNT. As shown in Table 7, low-dose proteins (0.025 mg/kg of TL-16-h1-hFc or TL-25-h1-hFc; 0.05 mg/kg of TL-34-h1-hFc) remained effective up to 24 h after exposure. Treatment of mice in the early stage of toxin exposure, especially before the onset of symptoms, was the best way to protect them against exposure to TeNT.

In conclusion, our results demonstrate that the three humanized VHH-hFc fusion proteins conferred protective effects against TeNT in a mouse model, with efficacy dependent on both dose and timing of administration.

## 4. Discussion

The TeNT L domain is an active protease; the HN domain is the toxin translocation domain; and the Hc domain contains the receptor-binding domain. Previous studies have mainly focused on the Hc domain as an important target for antibody development because of the presence of key protective epitopes [34,35,36,37,38,39,40]. However, the use of only a single L or HN domain might result in some epitopes being missing or not recognized [41,42,43]. Our previous study also showed that the TL-HN functional fragments provided the best immunoprotection among all TeNT fragments [28], which was also observed in studies of BoNT/E and F (toxins with similar structures and modes of action to TeNT) [32,33]. This might be because the folding of the L and HN domains forms novel neutralizing antibody epitopes that enhance immunogenicity, similar to the functional alignment of chaperones between the L and HN domains of BoNTs [44].

Recent studies have suggested that antibodies targeting not only Hc but also L + HN and L are able to neutralize TeNT [28]. For example, Vilk et al. showed that a monoclonal antibody (mAb) provided protection against tetanus target epitopes located throughout the TeNT molecule [41]. Most of the neutralizing antibodies present in commercial polyclonal anti-TeNT antibodies were found to bind to L in a study by Lang et al. [40]. In our study, a phage-display VHH library was constructed by immunizing a camel with L-HN. Thirty-six VHHs against TL-HN were identified from the library. After fusion with hFc and humanization, three humanized VHH-hFc fusion proteins (TL-16-h1-hFc, TL-25-h1-hFc, and TL-34-h1-hFc) were constructed with high affinity and neutralizing activity, a prolonged half-life, and minimized adverse immunogenic side effects.

We demonstrated that TL-16-h1-hFc and TL-25-h1-hFc require only 0.3125 μg to completely neutralize 10 × LD_50_ TeNT. Moreover, 125 μg/kg of TL-16-h1-hFc or TL-25-h1-hFc could provide complete protection to mice within 12 days. In addition, 25 μg/kg of TL-16-h1-hFc or TL-25-h1-hFc remained effective up to 24 h after exposure to TeNT.

In a study by Aliprandini et al., the neutralizing potency of a number of antibodies conjugated to different structural domains was tested. Among them, a combination of three monoclonal antibodies reacting with the L or HN structural domains provided complete protection to all mice from any signs of toxicity or death [43]. In Lukić’s study, it was found that of the eight antibodies, only Mab51 (which binds to the light chain) provided complete protection to mice [45]. In our previous study, the chimeric heavy-chain antibody T83-13 targeting THC required 1 mg/kg to provide complete protection against 20 × LD_50_ TeNT [35]. In contrast, our humanized VHH-hFc fusion proteins showed a very high neutralization capacity compared with antibodies against other epitopes.

Moreover, combining multiple mAbs targeting different epitopes of the same antigen can significantly increase the protective effect. In Yi Li et al.’s study [39], antibody 4C1 provided complete protection at 10 × LD_50_ when used alone with 200 μg of antibody. The combination of antibodies 3A7 and 4C1 at a dose of 20 μg (10 μg + 10 μg) resulted in complete protection from toxin attack at 10 × LD_50_ (6 μg + 6 μg + 6 μg) and achieved complete neutralization of the toxin at 10 × LD_50_. The effects of the combination therapy were compared with those of the individual mAbs, which confirmed the superior protection of the combination therapy. The results suggest that the combination of antibodies targeting non-overlapping antigenic epitopes enhances the neutralizing effect. We observed similar effects: we combined TL-25-h1-hFc and T92-6-hFc-h2 (targeting THC), which provided comprehensive protection at lower doses (unpublished data), offering great potential for the subsequent construction of bispecific antibodies.

This study successfully identified three high-affinity anti-TeNT humanized VHH-hFc fusion proteins and demonstrated that the combined application of two or three humanized VHH-hFc fusion proteins offers a synergistic effect. The current investigation offers a platform for future pharmacological development against TeNT.

## Figures and Tables

**Table 1:** TL-HN phage VHH library panning enrichment analysis.

Panning Round	Input	Output	Output/Input Ratio	Enrichment Factor
First	5.0 × 10^11^	4.2 × 10^7^	8.4 × 10^−5^	-
Second	1.0 × 10^11^	1.95 × 10^7^	1.95 × 10^−4^	2.3

**Table 2:** Neutralizing potency of the VHH-hFc fusion proteins against TeNT.

Group ^a^	Dose of TeNT ^b^	Surviving/Total Mice ^c^	Survival (%)	Group ^a^	Dose of TeNT ^b^	Surviving/Total Mice ^c^	Survival (%)
TL-1-hFc	10 × LD_50_	0/4	0	TL-19-hFc	10 × LD_50_	0/4	0
TL-2-hFc	10 × LD_50_	0/4	0	TL-20-hFc	10 × LD_50_	0/4	0
TL-3-hFc	10 × LD_50_	0/4	0	TL-21-hFc	10 × LD_50_	2/4	50
TL-4-hFc	10 × LD_50_	0/4	0	TL-22-hFc	10 × LD_50_	0/4	0
TL-5-hFc	10 × LD_50_	0/4	0	TL-23-hFc	10 × LD_50_	0/4	0
TL-6-hFc	10 × LD_50_	0/4	0	TL-24-hFc	10 × LD_50_	0/4	0
TL-7-hFc	10 × LD_50_	0/4	0	TL-25-hFc	10 × LD_50_	4/4	100
TL-8-hFc	10 × LD_50_	0/4	0	TL-26-hFc	10 × LD_50_	0/4	0
TL-9-hFc	10 × LD_50_	0/4	0	TL-27-hFc	10 × LD_50_	0/4	0
TL-10-hFc	10 × LD_50_	0/4	0	TL-28-hFc	10 × LD_50_	0/4	0
TL-11-hFc	10 × LD_50_	0/4	0	TL-29-hFc	10 × LD_50_	0/4	0
TL-12-hFc	10 × LD_50_	2/4	50	TL-30-hFc	10 × LD_50_	3/4	75
TL-13-hFc	10 × LD_50_	0/4	0	TL-31-hFc	10 × LD_50_	1/4	25
TL-14-hFc	10 × LD_50_	0/4	0	TL-32-hFc	10 × LD_50_	2/4	50
TL-15-hFc	10 × LD_50_	0/4	0	TL-33-hFc	10 × LD_50_	0/4	0
TL-16-hFc	10 × LD_50_	4/4	100	TL-34-hFc	10 × LD_50_	4/4	100
TL-17-hFc	10 × LD_50_	0/4	0	TL-35-hFc	10 × LD_50_	0/4	0
TL-18-hFc	10 × LD_50_	0/4	0	TL-36-hFc	10 × LD_50_	0/4	0
Dilution buffer	10 × LD_50_	0/4	0				

^a^ Mice were injected with 5 μg VHH-hFc fusion protein, respectively. ^b^ KM mice were injected intraperitoneally (i.p.) with the mixture of 10 × LD_50_ of TeNT and the VHH-hFc fusion protein, respectively. ^c^ Data represent the number of mice that remained alive (survivors).

**Table 3:** Dissociation constants of the VHH-hFc fusion proteins.

Protein	Kon (10^4^ Ms^−1^)	Kdis (10^−4^ s^−1^)	KD (nM)	χ^2^	R^2^
TL-16-hFc	2.38	2.39	10.1	0.579	0.99
TL-25-hFc	2.59	4.44	17.1	0.0198	0.99
TL-34-hFc	4.66	1.68	3.61	0.6906	0.99

All data were calculated using a 1:1 binding model in Analysis Software 7.0. Kon, association constant; Kdis, dissociation constant; KD, equilibrium dissociation constant; KD, Kdis/Kon.

**Table 4:** Dissociation constants of the humanized VHH-hFc fusion proteins.

Protein	Kon (10^4^ Ms^−1^)	Kdis (10^−4^ s^−1^)	KD (nM)	χ^2^	R^2^
TL-16-h1-hFc	2.239	0.2036	0.9094	0.2084	0.99
TL-25-h1-hFc	1.89	0.109	0.573	0.5898	0.99
TL-34-h1-hFc	3.98	0.252	0.633	0.6744	0.99

All data were calculated using a 1:1 binding model in Analysis Software 7.0. Kon, association constant; Kdis, dissociation constant; KD, equilibrium dissociation constant; KD, Kdis/Kon.

**Table 5:** Neutralizing potency of the combination of humanized VHH-hFc fusion proteins against TeNT.

Group	Dose of Protein ^a^ (μg)	Dose of TeNT ^b^	Surviving/Total Mice ^c^	Survival (%)
TL-16-h1-hFc + TL-25-h1-hFc	0.156 + 0.156	10 × LD_50_	4/4	100
	0.078 + 0.078	10 × LD_50_	4/4	100
	0.039 + 0.039	10 × LD_50_	3/4	75
	0.0195 + 0.0195	10 × LD_50_	0/4	0
TL-16-h1-hFc + TL-34-h1-hFc	0.156 + 0.312	10 × LD_50_	4/4	100
	0.078 + 0.156	10 × LD_50_	4/4	100
	0.039 + 0.078	10 × LD_50_	4/4	100
	0.0195 + 0.039	10 × LD_50_	2/4	50
TL-25-h1-hFc + TL-34-h1-hFc	0.156 + 0.312	10 × LD_50_	4/4	100
	0.078 + 0.156	10 × LD_50_	4/4	100
	0.039 + 0.078	10 × LD_50_	4/4	100
	0.0195 + 0.039	10 × LD_50_	3/4	75
TIG	0.1 IU	10 × LD_50_	4/4	100
Dilution buffer	-	10 × LD_50_	0/4	0

^a^ Mice were injected with different doses of the humanized VHH-hFc fusion protein groups. ^b^ KM mice were injected intraperitoneally (i.p.) with 10 × LD_50_ of TeNT and humanized VHH-hFc fusion protein groups mixture, respectively. ^c^ Data represent the number of mice that remained alive (survivors).

**Table 6:** Prophylactic efficacy of the humanized VHH-hFc fusion proteins against TeNT in a mouse model.

Antibody ^a^	Dose of Protein ^b^	Dose of TeNT ^c^	Number of Survivors/Total Mice per Group								
			12 h ^d^	24 h	48 h	3 d	5 d	7 d	9 d	12 d	14 d
TL-16-h1-hFc	25 μg/kg	10 × LD_50_	4/4 **	4/4 **	4/4 **	2/4 **	0/4 **	0/4 **	0/4 **	0/4 **	0/4 **
	125 μg/kg	10 × LD_50_	4/4 **	4/4 **	4/4 **	4/4 **	4/4 **	4/4 **	4/4 **	4/4 **	3/4 **
TL-25-h1-hFc	25 μg/kg	10 × LD_50_	4/4 **	4/4 **	4/4 **	4/4 **	3/4 **	1/4 **	1/4 **	0/4 **	0/4 **
	125 μg/kg	10 × LD_50_	4/4 **	4/4 **	4/4 **	4/4 **	4/4 **	4/4 **	4/4 **	4/4 **	3/4 **
TL-34-h1-hFc	50 μg/kg	10 × LD_50_	4/4 **	4/4 **	3/4 **	2/4 **	0/4 **	0/4 **	0/4 **	0/4 *	0/4 **
	250 μg/kg	10 × LD_50_	4/4 **	4/4 **	4/4 **	4/4 **	4/4 **	4/4 **	2/4 **	0/4 **	0/4 **
TIG	0.1 IU	10 × LD_50_	4/4 **	4/4 **	4/4 **	4/4 **	4/4 **	4/4 **	4/4 **	4/4 **	4/4 **
B-h3	250 μg/kg	10 × LD_50_	0/4	0/4	0/4	0/4	0/4	0/4	0/4	0/4	0/4
PBS	-	10 × LD_50_	0/4	0/4	0/4	0/4	0/4	0/4	0/4	0/4	0/4

^a^ Mice exposed to TeNT were injected intravenously (i.v.) with TL-16-h1-hFc, TL-25-h1-hFc, TL-34-h1-hFc, TIG, B-h3, and PBS at the indicated times before exposure. ^b^ TeNT-exposed mice were injected with different doses of the humanized VHH-hFc fusion proteins, namely 0.1 IU TIG, 250 μg/kg B-h3, or PBS, at the indicated times before exposure. ^c^ KM mice were injected intraperitoneally (i.p.) with 10 × LD_50_ of TeNT. ^d^ Mice were treated i.v. with the indicated doses of the humanized VHH-hFc fusion proteins at 12 h, 24 h, 48 h, 3 d, 5 d, 7 d, 9 d, 12 d, and 14 d before exposure to TeNT. Data represent the number of mice that remained alive (survivors). The statistical analysis was performed using the log-rank test of GraphPad Prism 8 software (** *p*  <  0.01; * *p*  <  0.05).

**Table 7:** Therapeutic efficacy of the humanized VHH-hFc fusion proteins against TeNT in a mouse model.

Antibody ^a^	Dose of Protein ^b^	Dose of TeNT ^c^	Number of Survivors/Total Mice per Group				
			1 h ^d^	3 h	6 h	12 h	24 h
TL-16-h1-hFc	25 μg/kg	5 × LD_50_	4/4 **	4/4 **	4/4 **	4/4 **	4/4 **
	125 μg/kg	5 × LD_50_	4/4 **	4/4 **	4/4 **	4/4 **	4/4 **
TL-25-h1-hFc	25 μg/kg	5 × LD_50_	4/4 **	4/4 **	4/4 **	4/4 **	4/4 **
	125 μg/kg	5 × LD_50_	4/4 **	4/4 **	4/4 **	4/4 **	4/4 **
TL-34-h1-hFc	50 μg/kg	5 × LD_50_	4/4 **	4/4 **	4/4 **	4/4 **	4/4 **
	250 μg/kg	5 × LD_50_	4/4 **	4/4 **	4/4 **	4/4 **	4/4 **
TIG	0.1 IU	5 × LD_50_	4/4 **	4/4 **	4/4 **	4/4 **	4/4 **
B-h3	250 μg/kg	5 × LD_50_	0/4	0/4	0/4	0/4	0/4
PBS	-	5 × LD_50_	0/4	0/4	0/4	0/4	0/4

^a^ Mice exposed to TeNT were intravenously (i.v.) treated with TL-16-h1-hFc, TL-25-h1-hFc, TL-34-h1-hFc, TIG, B-h3, and PBS at the indicated times after exposure. ^b^ TeNT-exposed mice were treated with different doses of the humanized VHH-hFc fusion proteins, namely 0.1 IU TIG, 250 μg/kg B-h3, or PBS, at scheduled times after exposure. ^c^ KM mice were injected intraperitoneally (i.p.) with 5 × LD_50_ of TeNT. ^d^ Mice were treated i.v. with the indicated doses of the humanized VHH-hFc fusion proteins at 1 h, 3 h, 6 h, 12 h, or 24 h after exposure to TeNT. Data represent the number of mice that remained alive (survivors). The statistical analysis was performed using the log-rank test of GraphPad Prism software (** *p*  <  0.01).

## Data Availability

The data that support the findings of this study are available from the corresponding author upon reasonable request.
